# T-cell Receptor Is a Threshold Detector: Sub- and Supra-Threshold Stochastic Resonance in TCR-MHC Clusters on the Cell Surface

**DOI:** 10.3390/e24030389

**Published:** 2022-03-10

**Authors:** László Bene, Miklós Bagdány, László Damjanovich

**Affiliations:** 1 Department of Surgery, Faculty of Medicine, University of Debrecen, 4032 Debrecen, Hungary; dami1960@med.unideb.hu; 2 Department of Physiology, McGill University, Montreal, QC H3A 0G4, Canada; miklos.bagdany@mcgill.ca

**Keywords:** noncognate–cognate discrimination, MHCI/MHCII, TCR, CD8/CD4, distributed signal detection, chimeric antigen receptor (CAR) optimization, mutual information, correlation, pattern recognition, Förster resonance energy transfer (FRET)

## Abstract

Stochastic resonance in clusters of major histocompatibility molecules is extended by a more detailed description of adaptive thresholding and by applying the notion of suprathreshold stochastic resonance as a stochastically quantizing encoder of transmembrane signaling downstream of major histocompatibility molecules and T-cell receptors on the side of presenting and recognizing cells, respectively. The adaptive nature of thresholding is partly explained by a mirroring of the noncognate–cognate dichotomy shown by the T-cell receptor structure and the kinetic-segregation model of the onset of T-cell receptor triggering. Membrane clusters of major histocompatibility molecules and T-cell receptors on their host cells are envisioned as places of the temporal encoding of downstream signals via the suprathreshold stochastic resonance process. The ways of optimization of molecular prostheses, such as chimeric antigen receptors against cancer in transmembrane signaling, are suggested in the framework of suprathreshold stochastic resonance. The analogy between Förster resonance energy transfer and suprathreshold stochastic resonance for information transfer is also discussed. The overlap integral for energy transfer parallels the mutual information transferred by suprathreshold stochastic resonance.

## 1. Introduction

Molecular associations of immune receptors organized around the MHCI and MHCII molecules have been observed on the nm-μm distance scales in the past by using biophysical techniques such as FRET, confocal microscopy, AFM, and TEM [1,2,3,4,5]. Supramolecular complexes of MHCI, MHCII, ICAM-1, transmembrane-4, or tetraspan (TM4SF) molecules, transferrin receptor, and the interleukin-2 and -15 receptor subunits (α, β, γ_c_) have been found on the surface of several cancerous cell lines, as well as on the CD4^+^ T-cells from the peripheral blood and lymph nodes of Crohn’s diseased persons [1,2,3,4]. Although these observations fit well into the current concept of lipid-rafts as a membrane-organizing paradigm, the specific role of the MHCI and MHCII molecules in these clusters has been left largely enigmatic [5]. Besides their conventional roles in immune-recognition, the mechanical stabilization of lipid rafts, as well as their “non-classical” roles in signal-transduction, has been suggested [6,7,8,9].

Next, we show that the combination of the lipid raft concept with that of the stochastic resonance (SR) phenomenon gives rise to a functional framework in which the observed geometrical rearrangements of the MHC clusters and their intracellular associations can obtain deeper explanations, e.g., MHC and TCR clusters [10] can be envisioned as places of temporal encoding of downward signals via SR, i.e., as molecular encoders carrying out stochastic digitization.

Both TCR and MHC stand in the front line of adaptive immunity in the initiating immune processes for defending the human body from foreign invaders, such as viruses [11]. During immune recognition, TCRs on the surface of a T cell are capable of very high efficiency scanning of the surface of the APC, whereby the presence of only a few pMHCs (1–10 peptides/APC) on the surface of the APC might be adequate for the successful activation of the T cell. TCR signaling results in the elevation of intracellular Ca^2+^ and PKC levels. Furthermore, signaling is initiated with the involvement of ZAP70 towards the nucleus, where transcription of growth factors such as cytokines are initiated. We are asking for now the type of mechanisms that stand behind the afore-mentioned exquisite sensitivity. The initial contact of TCRs with pMHCs triggers a cascade on the surface of the T cell, leading to the formation of the immune synapse between the T cell and the APC, a junctional region resembling both in structure and function the synapse between nervous cells. However, in cross section, this junctional region is not homogeneous, with concentric circular regions called “central supramolecular activation cluster (SMAC)”, (cSMAC), being in the middle, peripheral SMAC (pSMAC), circumventing the middle, and distal SMAC (dSMAC), circumventing the previous two [11]. During maturation of the synapse, TCRs move centripetally inward, from the “d” towards the “c” region, with their molecular neighbourhood signaling activity changing in parallel. In the early phase of activation corresponding to the dSMAC region, TCRs are monomers and quickly scan the surface of the APC for an antigen. Upon meeting with antigen, activation begins and microclusters of TCRs are formed. TCR microclusters are then translocated through the pSMAC zone, forming larger clusters of TCR in the cSMAC region, where they are internalized (endocytosed) to ensure TCR turnover. If on the surface of a killer T cell, the cSMAC is supposedly also the place where agents of lytic activity are ejected towards the APC.

In our work, we consider TCR in the first and second phase of signaling corresponding spatially to the dSMAC and pSMAC regions of the gradually evolving (maturing) IS, with gradually growing homo-clusters of TCR [11]. Based on literature data, we give reasoning for the existing thresholds already at the single molecule level for the TCR, on which building a functional model based on SR is proposed. Furthermore, these single molecular thresholds, if completed with possible modulations by molecular partners (co-receptors) in the different association forms, kinases, and physiological states of the embedding lipid rafts, might also be considered as cores for a threshold hierarchy [12] for a panel of different functions, e.g., eliciting the timely production of different growth factors (cytokines) and other biological activities.

SR, as a dynamic process, was discovered at the beginning of the 1980’s by Benzi and Nicolis [13,14]. They explained the recurrence period of c.a. 10^5^ years of ice ages with a superposition of a periodic change of the Earth-Sun orbit eccentricity due to galactic attraction as a weak periodic process and random irradiance fluctuations of the Sun due to, e.g., the Sun’s or volcano’s eruptions, constituting a quickly fluctuating “noise”. Upon the addition of “noise”, an otherwise subthreshold (“not detectable”) signal becomes above-threshold (“detectable”), enabling the given process to take place. This so-called dynamic stochastic resonance can also be envisioned by the Eyring’s transition rate theory, according to which the barrier crossing rate of a ball in a periodically shaking double-well potential, such as two baskets, is enhanced whenever the shaking frequency matches that of the ball’s vibration in the wells [15,16]. Later, in 1995, nondynamical SR, when a superposing noise lifts a signal above the threshold, was discovered by Gingl and Moss [17,18]. In the meantime, a plethora of phenomena realizing some form of SR in both the living world, as well as in the artificial-natural non-living world have been discovered, ranging from the workings of the sensory systems of humans, animals, and neural networks to distributed detection, artificial pattern recognition, and social sciences [19,20,21,22,23,24,25,26,27].

A new kind of SR was discovered by N.G. Stocks, at the beginning of the 2000’s, called “suprathreshold stochastic resonance,” and dubbed SSR [28,29,30]. He realized that by parallel connecting a number of threshold detectors (‘threshold devices’), each capable for the conventional subthreshold SR, a stochastic resonance can be observed at the output of this detector array at changing the noise levels on the detectors, independently from the size of the input signal, i.e., whether it is sub- or supra-threshold. However, this new SR occurs not in the “signal-per-noise ratio” (SNR) as for the conventional subthreshold “single-device” SR, but in “appropriately defined” parameters borrowed from the realm of information theory, such as mutual information, channel capacity, coding efficiency, mean distortion error, and input-output signal correlation [31]. The majority, if not all, of these parameters have been shown to monotonously increase (or decrease) with an increasing number of detectors and to possess a definite maximum (or minimum) as a function of noise level, almost independently of the actual type of statistics of noise and input signal (i.e., whether they are Gaussian, Poissonian, etc.). Furthermore, he and his co-workers also show that these parallel connected devices essentially behave as stochastic signal digitizers [31]. What is remarkable is also that the existence of the above-mentioned maxima (or minima) indicates that the encoding functionality can be optimised by a careful engineering of the array structure (analogously to the cluster morphology of receptors), i.e., the number of detectors and the amount of injected (or inherent) “noise”.

By identifying cluster elements with the corresponding ingredients of SR, fixation of molecular impairments in different illnesses, i.e., therapeutic interventions, might become more readily realisible. This interpretation of the role of receptor clustering may also help in the optimisation of engineered molecular prostheses such as “chimeric antigen receptors” (CARs) [32,33,34,35,36,37], which are intended to improve TCR functionality, similarly to the optimisation of cochlear implants by SR to improve hearing [38].

Akin to any sensory function, such as hearing, immune sensing can also be damaged. For example, in cancer, MHC levels on the surface of APC can be downregulated, or the stimulatory potential (antigenicity) of the presented peptide can be weak, both implying escaping of cancerous cells from the servailing killing T cells. Bypassing MHC restriction might be a remedy by expressing genetically engineered, artificial, TCR-like receptors, called “chimeric antigen receptors” (CARs), on the effector T cells of patients [32]. Here, the main problem is the optimized tuning of the new receptors via their available signaling (phosphorylatable) sites while also appropriately taking into account the local molecular milieus of the T cells and APCs of the patient. In the case of weak CAR, the system might not be adequate to eliminate cancer, whereas for strong CAR, overshooting might occur, manifesting in a “cytokine storm,” which might be even fatal for the patient. In the case of hearing, the “local molecular milieu” is represented by the Brownian motion of the tip of hair cells as the “noise” and assists in hearing via the SR process. According to experience with cochlear implants, by artifically stimulating auditory nerve cells by introducing electric noise at the level of Brownian motion as the “noise”, sound recognition has improved [31,38]. An important feature, however, of this observation was the similar (or almost equal) magnitudes of the signal and noise for SR to occur. As to the TCR (or TCR-like) systems, both the signal and noise are measured by the number of phosphorylatable sites.

As to the molecular structure, all CARs consist of an ectodomain, a transmembrane domain, and an endodomain [33]. The farthest, most distal from the membrane, portion of the ectodomain is a signal peptide, which drives the nascent protein into the endoplasmic reticulum. Connected to this is an scFv region composed of the antigen-recognizing variable portions of the light and heavy chains of an antibody. The scFv region is connected to the transmembrane region, an alpha helix spanning the membrane, through a linker, the hinge region of an IgG_1_ antibody. The endodomain is the signaling region, which is the signaling domain of CD3ζ, comprising of three “tyrosine-based activation motifs” (ITAMs) [33]. Upon engagement with antigen, CARs cluster and signaling is initiated towards the nucleus. In the original, first generation of CAR, the CD3ζ is alone (symbolized by scFv-CD3ζ). In the second generation, CD3ζ is extended with the signaling domain of the co-receptor CD28 (symbolized by scFv-CD28-CD3ζ). In the third generation, the signaling domain of the second generation is extended with a mixture of signaling domains of CD134 and CD137 (symbolized by scFv-CD134/CD137-CD28-CD3ζ). In the fourth generation, the CD28 signaling domain of the third generation is replaced by the signal domain of interleukin-12 (IL-12) (symbolized by scFv-CD134/CD137-IL-12-CD3ζ).

Besides conventional CARs, there also exists a genetically engineered TCR (TCR:ζ), which is a heterodimer of the TCRα and β chains as the ectodomain, connected to a complete CD3ζ signal chain as the endodomain. The important observation is that the TCR:ζ function is optimal when TCR:ζis incorporated into the wild-type TCR-CD3ζ complex [34,36]. This also stresses the importance of the homo-associations of the TCR-like receptors in signaling.

Concerning our present work, the relevance of SSR in immunology might be raised by the following issues:

(i) The resemblance of immune system to the neural system. Information is exchanged between interactions of circulating cells or populations thereof, e.g., in the case of immune recognition via the immune synapses (ISs) between antigen-presenting cells (APCs) and recognizing helper or killer T-cells with the aid of noncognate- and cognate-peptide fragments of 8–9 amino acids long, acting similarly to the neurotransmitters [39,40]. The process between the circulating cells can be likened to that between a pair of single nerve cells or two collections of them participating in the “neural network”, the traditional places of both sub- and supra-threshold SRs. In the contact zone between the APC and T-cell, an array of MHCs present the noncognate- and cognate-peptides acting as ‘neuro-transmitters’ to the recognizing single TCR molecules or their arrays. In the case of a malfunction of this antigen-recognizing system, molecular restructuring might be easier in terms of an established functional framework, such as SR or SSR.

(ii) Operation of the immune system should also take place in suboptimal, “noisy” conditions, as meant by the different diseases (e.g., cancerous, auto-immune). This implies the relevance of the single-detector sub-threshold SR, which has been proven to be effective in suboptimal situations. In cancer, SSR might also be observable at a cell population level [38,40,41,42]. Involvement of SR in immune recognition by the TCR-MHC system might also be justified by the observation that activation of the immune system has been elicited by just a few peptide-loaded MHCs (1–10 peptides/APC). In the context of immune recognition, SR was first mentioned in [43,44] as an explanation for TCR discriminating between the noncognate- and cognate-peptides presented by MHC. Experiments consistent with SR have been reported in [45,46,47,48]. Theoretical modelling of ligand discrimination by TCR has also been carried out in [49,50]. When compared to these works, SSR might have the advantage of enabling optimization of the elements of SSR-like “noise”, thresholds, and coreceptors, by comparing the efficiencies of information transfer through the detector array. The efficiency of information transfer can be quantitated, e.g., with the relative difference between the information content of the output and input signals. These features of SSR might be advantageously utilised to optimize CAR systems for invading cancer. In [51], dealing with SR from the viewpoint of MHC, additional experimental reports consistent with SR have been listed.

(iii) The possible needs for the optimisation of artificial, engineered molecular recognition systems such as chimeric antigen receptors (CARs) [32,33,34,35,36,37], which can be more purposefully performed in the framework of SR and SSR [31].

(iv) Intercellular communication via cell contacts is a pattern-recognition process. SR is a special pattern recognition process [24,25].

However, to accomplish these tasks, first the main ingredients of SR and SSR, the nature of the input and output signals, the processes with the role of noise, and the structure and mechanism realizing the thresholding act, should be assigned tot he immunological signalling processes at hand.

## 2. Properties of the TCR-MHC System Analyzed from the Viewpoint of SR

Next, based on the structural and conformational dynamical data found in the literature, we propose that TCR is a threshold detector. This fact alone already indicates that SR processes might be utilized by the cell in immune recognition, and it makes the occurrence of some type of SR highly probable. Recipes for experimental justification are recommended in [51].

### 2.1. TCR Behaves as a Threshold Detector

#### 2.1.1. Structure and Signaling of TCR

In the following, we give a putative model of TCR signaling induced by engagement with peptide-loaded MHC (pMHC) molecules. The model has been based on literature data observed in other laboratories, with our intension to envision the conformational changes of TCR also conceivable as thresholds. With time, some fine details, e.g., of the precise role of coreceptors CD4 and CD8, might be proven to be incorrect—stabilizing the TCR-pMHC complex and/or assigning enzymatic activity to TCR by recruiting the Src tyrosine kinase p65lck (Lck), but the essence, which is the existence of a signal threshold for TCR, should remain valid.

**Figure 1:**
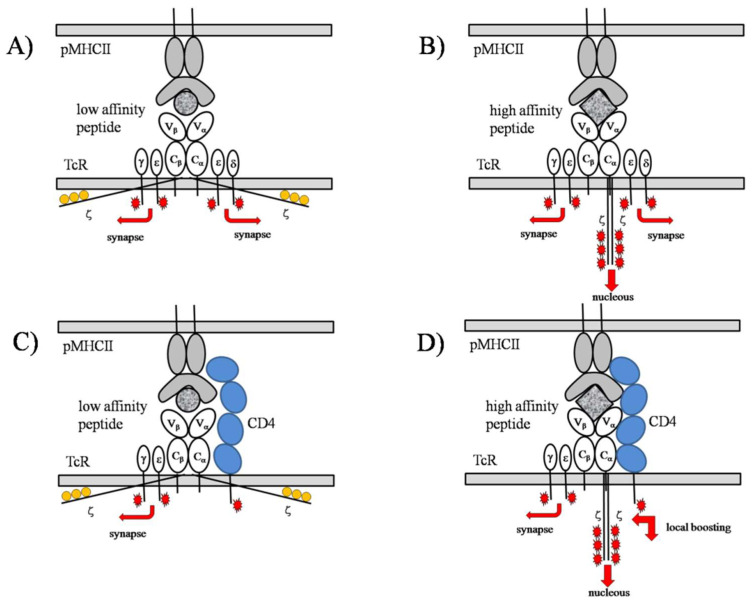
*TCR structure mirrors noncognate–cognate dichotomy.* Structural proof that TCR is a threshold detector. Panel **A**: In the first phase of TCR triggering, TCRs scan the surface of the APC without coreceptors. Panel **B**: Although in the first phase mainly low-affinity pMHCII bind, sometimes, dictated by the relative abundance, high-affinity or cognate pMHCII (cognate ligand), shaded square, can also bind. Panel **C**: During the second phase of TCR triggering, TCR scanning happens in the delimited spatial region called the immune synapse (IS), in the presence of a coreceptor (CD4, in blue) in close proximity to TCR. Panel **D**: In the second phase of TCR triggering, the signaling is mainly due to the binding of high-affinity, cognate pMHCII.

According to the scheme of the signaling mechanism of TCR, depicted in Figure 1, mechanical forces leading to conformational changes of the subunits play a decisive role in the initiation of signals, which are further modulated by molecular associations with neighboringTCRs, the presence of coreceptors, kinases, and phosphatases, and the size and morphology of the accommodating lipid raft. These conformational changes also define the thresholds via their free energy requirements (∆G) to take place, similarly to e.g, ion channel gating.

Panel A: In the first phase of TCR triggering, TCRs scan the surface of the APC without coreceptors. The purpose of this phase is the spatial gating or focusing on the presenting MHC cluster in the contact zone, imprinting the morphology of MHC clusters on APC to that of TCRs on T-cells by gradual segregation of the spatial delimiter adhesion molecules (LFA-1, CD28) and phosphatases (CD45, CD148), and bringing them into the contact zone of the coreceptors (CD4/CD8). Molecules are moved by the cytoskeleton as a response to signals elicited by the binding of both noncognate and cognate pMHCs by the TCR in the absence of coreceptors [52,53,54,55,56]. Binding of low affinity, or noncognate peptide, shaded circle, pMHCII leads to short-range and brief conformational changes of TCR involving only the ectodomains and culminating signals, red arrows, spreading parallel to the membrane surface and restricted to membrane proximal zones. In this process, only the tails of the γε and δε subunits will separate from the inner surface of the membrane and their ITAMs will be phosphorylated, red stars, by proximal Lcks, and those of the ζζ—tails with yellow circles—will remain sticking to it and not be phosphorylated [57,58,59,60,61]. Panel B: Although in the first phase, mainly low-affinity pMHCII binds, sometimes, dictated by the relative abundance, high affinity or cognate pMHCII (agonist, or cognate ligand), shaded square, can also bind. In this case, the elicited conformational change is more profound and long-lasting, culminating in the phosphorylation of ITAMs on the ζζ subunit in addition to those of the γε and δε subunits. The rate-limiting step of this conformational change is when the membrane stalk of the C_α_ domain becomes juxtaposed with those of the two ζ chains, reminiscent of a gear controller [62]. Upon this action, the ζ chains will be freed from the membrane surface and exposed to the phosphorylating Lcks. Another facet of this conformational change is the catch bond formation, which leads to lengthening of the conformational change [62]. However, in contrast to the γε and δε subunits when the signal spreads mainly parallel and close to the membrane surface, with the ζ chains the signal spreads towards the nucleus, meaning a real activation signal. Panel C:: During the second phase of TCR triggering, TCR scanning happens in the delimited spatial region called the immune synapse (IS), in the presence of a coreceptor (CD4, in blue), in close proximity to the TCR. The presence of coreceptor is for tuning TCR binding towards high-affinity pMHCs. In the presence of a coreceptor, low-affinity pMHC binding becomes obstructed by spatial hindering of pMHC binding due to restricted orientational flexibilities, ‘local cooling’ before binding, and the short residence time after binding. δε subunit signaling is not shown here, for clarity. Panel D: In the second phase of TCR triggering, signaling is mainly due to the binding of high-affinity, cognate pMHCII. Here, the high-affinity pMHCII binding in the presence of CD4, the conformational change, and elicited signaling of TCR learned in the absence of coreceptor (Panel B), should be completed with a local signal amplification elicited by the intracellular domain of CD4 upon a conformational rearrangement of CD4 induced by the catch bond formed between the TCR and pMHCII [63,64]. In this conformational rearrangement, CD4 becomescloser to TCR. As a consequence, the shielding of low-affinity pMHC binding and the lengthening of high-affinity pMHC binding became more perfect. Additionally, Lcks hooked to the intracellular domain of CD4 readily phosphorylate the newly exposed ITAMs in close proximity, as an amplifying step (marked by double red arrow making a right angle). With this built-in amplification step, the TCR-pMHC-coreceptor complex constitutes a threshold amplifier. That high-affinity pMHC binding is not affected so strictly by the spatial hindrance of CD4 as the low-affinity pMHC is performed, is reasonable, because of the induced fit phenomenon operating on the part of high-affinity pMHC, following also from the presence of catch bonds [65,66].

Additional markings: TCR: white ovals, peptide-MHCII complex (pMHCII): gray-shaded ovals, low-affinity (noncognate) peptide: gray-shaded circle, high-affinity (cognate) peptide: gray-shaded squares.

#### 2.1.2. The Noncognate–Cognate Dichotomy Is Mirrored by the TCR Structure

The first step of the initiation of the immune response is the first encounter of a TCR on the effector T cell with a peptide carrying MHC on the opposing APC surface at the point of first contact [52]. This step necessitates the long accessorial phosphatases CD45 and CD148, which are responsible for keeping phospho-kinase levels low, and adhesion molecules CD28, LFA-1, and LFA-3, creating the first contact, originally being in the vicinity of theTCR, to gradually segregate into distal points from the TCR [53,54]. These molecules act as spatial delimiters with the task of spatial gating on the area of APC covered by the peptide presenting MHC lattice, i.e., the MHC cluster size [4]. During this process, the baseline phospho-kinase level is also gradually elevated. In the meantime, TCR samples a number of peptide-loaded MHCs (pMHCs) regardless of whether the visited peptide (“p”) is of noncognate or cognate type.

The very basic signal encoding act is a conversion of the affinity of bound peptide to phosphorylated signaling segments, thirosine-containing activation motifs (ITAMs), of the TCR (marked by red stars on Figure 1). There are six ITAMs in TCR four pieces in γε and δε subunits sideways, and another six in the central ζζ subunit (Figure 1 Panel B). Importantly, the structure of TCR is organized in such a way to reflect the noncognate–cognate dichotomy already at the structural level [55]. Binding of low affinity ligands induces low amplitude, quick oscillations in the sideway domains γε and δε with enough energy to separate the four signaling tails, originally stacked on the inner leaflet of the membrane, from the shielded 2D state into an exposed 3D state, amenable for p56lck kinases to phosphorylate them (Figure 1 Panel A) [56,57,58,59,60]. In contrast to low affinity binding, affecting only a portion of TCR, high affinity binding leads to high-amplitude slow conformational oscillations affecting the whole molecule. A critical act is the transfer of the ζ chains from the original divaricated state into a final state in juxtaposition with the membrane-spanning stalk of the α-subunit (Figure 1 Panel B) [61,62,67]. This conformational rearrangement corresponds to the action of a gear-lever in switching the speed of a vehicle. This coupling act enables the large energy conformational oscillations to reach the ζ chains and separate them from the inner membrane leaflet, similarly to the case with the two sideways subunits, described above.

The closing act of conformational oscillations is always a liberation of some signaling tail from the shielded membrane-juxtaposed 2D state into an exposed 3D state (Figure 1 Panel B). The energy requirement of the liberation is dictated by the interaction with the membrane surface, which in turn is dictated by the local physiological state of the membrane, i.e., types of phospholipids, membrane/surface potential, cholesterol content, and dipole potential. For a closer illumination, at higher membrane potential the tails more strongly associate with the membrane via a stronger induced electric polarization effect than at low membrane potential. Additionally at higher membrane (and/or dipole) potential, the cell membrane is stiffer, which also opposes the above “gear-lever” effect.

From the above, we see that a first-level (or soft) threshold already exists, built into the structure of TCR. This threshold is also coupled with a different focusing of the evoked intracellular signals. Low-affinity binding induces phosphorylations to spread predominantly sideways (Figure 1 Panel A), remaining in membrane proximity, and ultimately activates processes regulating immune synapse (IS) formation by involving cortical regions of the cytoskeleton. As a result, spatial delimiter molecules (CD45, CD148, CD28, LFA-1, and LFA-3) move to the periphery of the immune synapse (IS), but coreceptors CD4 and CD8 move towards TCRs [68,69]. High-affinity binding induces phosphorylation in addition to those induced by low-affinity binding, spreading towards the cell nucleus as the real activating signals, ultimately resulting in gene transcription (Figure 1 Panel B). As a result of the scanning with a lonely TCR, or first stage of TCR triggering, an immune synapse is established on the T-cell as an imprint of the MHC cluster on the opposite cell (APC). In parallel, TCR is extended with the associating coreceptor CD4/CD8, with the purpose of refinement of the built-in threshold (Figure 1 Panels C,D). The extent and quality of these processes are governed by TCR signaling elicited by the binding of both low- and high-affinity peptides.

#### 2.1.3. TCR Triggering as a Biphasic Process: Adaptive Thresholding

We saw above that the first (or scanning) stage ends with the fixing of the spatial limits of the immune synapse and the appearing coreceptor CD4/CD8 on the side of TCR, aiming at a refinement of the built-in (or first) threshold (Figure 1 Panels C,D). A distinction between ligands i.e., specificity is achieved by spatially limiting the access of the ligands, peptide-holding MHC, pMHC, to the TCR. This spatial limitation results partly from reducing TCR and MHC flexibilities as a “local cooling” or “entropic stabilization” [47,70]. For example, the rotational freedom about the membrane-spanning stalk of MHCI in a plane parallel to the membrane and the rotational freedom of the stalk itself in planes perpendicular to the membrane are both severely hindered due to CD4 proximity [50]. Although the rotational flexibility of both noncognate and cognate peptide pMHCsis hindered sterically by CD4, cognate peptides can still bind via induced fit [65]. Peptide engagement of TCR has another benefit for improving noncognate–cognate discrimination: catch-bond formation of cognate peptides. Catch-bond formation means elongation of residence time in the TCR binding pocket via the peptide-induced rearrangements of TCR subunits adjacent to CD4/CD8, leading to stronger association of TCR and to CD4/CD8 and increased rigidity of the TCR-coreceptor complex with a further increase in specificity (Figure 1 Panel D) [63,65].

This sharpening of threshold implies a corresponding high-tuning of specificity in favor of high-affinity cognate pMHC. However, the increased specificity is concomitant with a reduction in sensitivity. As a compensation, CD4/CD8 has a built-in facility to enable in-situ local phosphorylation of the freed ζ chains via the hooked p56lck kinases, thereby increasing sensitivity [65]. Because of this ability, the TCR-CD4 complex behaves as a threshold amplifier. In this second phase of TCR triggering, scanning for pMHCs is performed by the different TCR-CD4 (or TCR-CD8) complexes separately, or in array format, as homo-associations. Homo-associations of TCR can be formed via the cytoskeleton. Forming homo-associations might be another way of increasing sensitivity, because of freeing, enabling it to be phosphorylated, the adjacent ζ chains of the associated TCRs [60,71,72,73,74].

### 2.2. SSR in TCR Clusters

We saw above that in the first phase of TCR triggering, scanning by the TCR happens basically without thresholding, with the purpose of teaching the TCRs to learn the level at which the threshold of detection is to be adjusted. Because of the overwhelming majority of the noncognate pMHCs, in almost all of the cases the nonactivating conformational pathway through the ectodomains of TCR is involved, leading to non-activating, weak signaling constrained mainly into the membrane proximal, cortical regions of the cytoplasm. Upon these signals, spatial filtering-out of noncognate pMHCs is carried out by the close positioning of coreceptor CD4/CD8 in apposition with TCR, thereby realizing a spatial gate of pMHC binding in the form of the TCR-CD4/CD8 complex, i.e., CD4/CD8 stabilizes the TCR-pMHC complexes. The gating efficiency is governed by the closeness of the TCR-CD4 proximity, which in turn is determined by the intracellular signal level learned by the TCR during free scanning.

**Figure 2:**
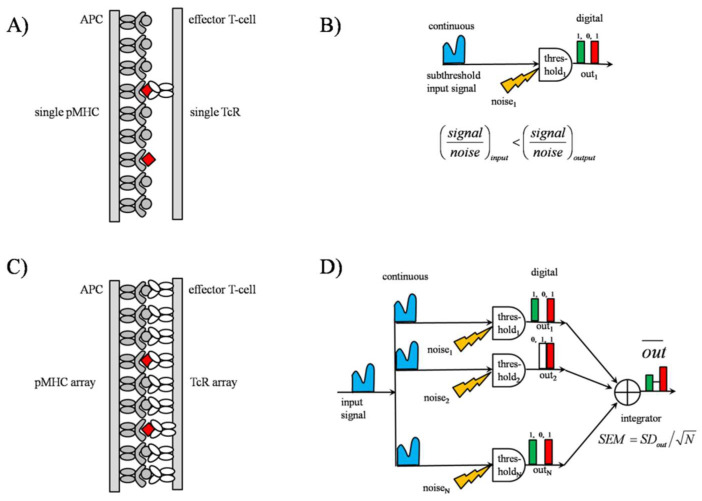
*Sub- and supra-threshold SR in TCR triggering.* Stochastic pooling network (SPN) model of the TCR array [28,29,30,31,71]. Panel **A**: Surface scanning by a single TCR. While in the first phase of triggering TCR detects both the noncognate and cognate peptides, in the second phase it detects mainly cognate ones by way of high tuning the threshold with coreceptor (not indicated) proximity. Threshold level has been adjusted based on the first phase detection. Because the threshold level has been adjusted to the net effects of both low- and high-affinity pMHCs, in the second phase, a portion of cognatepMHCs becomes subthreshold. For these pMHCs the subthreshold SR effect might be advantageously exploited to be detected. It is also visualized that for a single detector, the temporal behavior of output signal, red-white-green series of bars, might substantially differ from that of the input signal. Symbols: gray circle, noncognate peptide; red square, cognate peptide. Panel **B**: Schematic of single TCR scanning. In each moment, at the output a 0 (zero) or 1 (one) is assigned to the input, implying a 1-bit information transfer. Note that signal’s nature has been transformed from a continuous input to a digital output by SR. Panel **C**: Surface scanning by a TCR array. In the second phase of TCR triggering, although both the noncognate and cognate peptides are interrogated, only the cognate ones enable activating signals. Panel **D**: The equivalent pooling network model of the TCR array. Note that the signal’s nature has been transformed from a continuous input to a digital output by SSR.

In the second phase of TCR triggering, scanning is carried out by the TCR-coreceptor complexes realizing the spatial thresholding, either as lonely complexes or in homo-associated forms (Figure 2). Because the literature supports both forms, a mixed form can also be assumed. Homo-associations, “lattice-scanning”, speed up ligand finding and enhance sensitivity of detection, since the adjacent signaling chains of neighboring TCRs become the exposed form (Figure 2 Panels C,D). Because the threshold level is determined ultimately by the mixture of noncognate and cognate pMHCs in the first phase, a portion of cognate pMHCs can be expected to become subthreshold, in addition to the noncognate pMHCs, during the second phase of scanning. Consequently, the conventional subthreshold SR effect can be delayed for this portion of cognate pMHCs in the case of lonely scanning TCR-coreceptor complexes. As to the array scanning, SR effects occur irrespectively of the input signal size in the information content of the outgoing coded signal and correlation between the input and output by adjusting the noise.

#### 2.2.1. TCR Array as an “Equivalent Pooling Network”

The equivalent pooling network model of the TCR array is depicted in Panel D of Figure 2. At each moment, a zero or one appears on the output of each detector, which is summed up in the “integrator”. The network essentially carries out N+1-ary encoding of the input signal, with the output signal level n taking on possible values of zero, one, … N. While the single detector arrangement of Panel B can report only the presence or absence of an ”appropriate” input signal, the pooling network produces an output signal proportional to the size of the “appropriate” input signal. As a consequence, although the temporal profile of the output signal of the individual detectors might substantially deviate from that of the input signal, the temporal profile of the output signal produced by the “integrator” is highly correlated with that of the input signal. The effect is reminiscent of, but not the same as, that of averaging N independent measurement values for the same random variable, which reduces the error of the average (standard deviation of the mean, SEM) to $1/\sqrt{N}$-fold of the error of the individual measurements (standard deviation, SD): $SEM=SD/\sqrt{N}$.

#### 2.2.2. Signal Transmission by SSR

**Figure 3:**
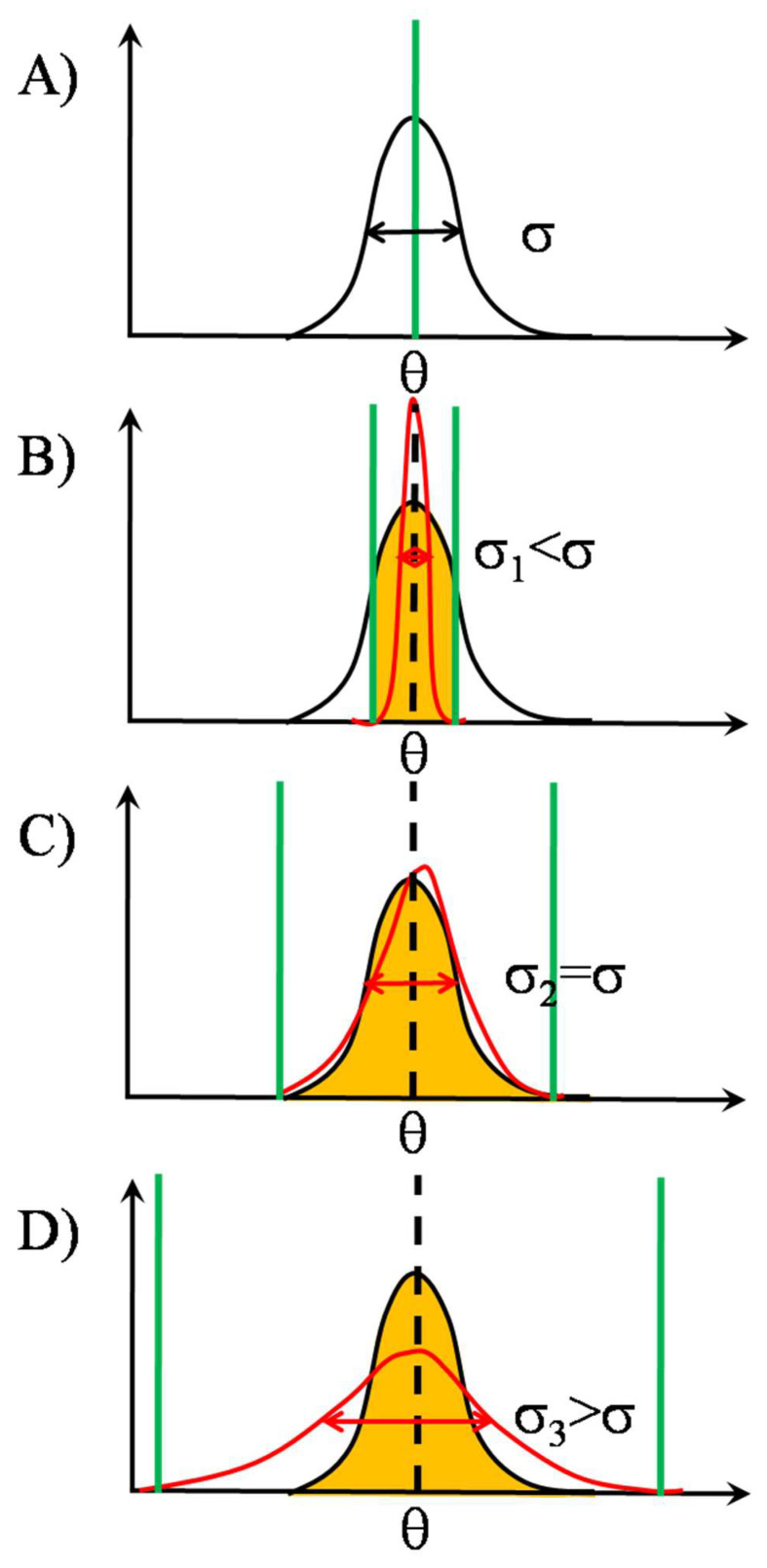
*Types of gating with threshold distributions. Mean-threshold gating with centered signal and threshold distributions.* Due to noise, the originally single-valued threshold (θ, marked by dashed vertical in black) becomes distributed (Gaussians marked by red colour), in the intervals delimited by the green vertical bars. The portions of signal distributions (Gaussians marked by black colour) affected by the threshold distributions are marked by orange shading. The net degree of influence is represented by the overlap regions of the threshold and signal distributions. Information transmission by the TCR array [31]. By opening out the threshold distribution, the intensity of sampling from the left, originally sub-threshold portion of the signal distribution is gradually increased at the cost of decreasing sampling from the right, originally supra-threshold portion. Panel **A**: Nominal threshold level of individual detectors in the TCR array of Figure 2 is designated by θ. Its position is marked by a vertical green thick line on the horizontal axis of the detected signal, whose distribution is represented as a Gaussian black curve with standard deviation (width parameter) σ. The detected signal can be the affinity of pMHC binding itself or any signal proportional to it, e.g., extent of conformational change in TCR, size or duration of force arisen between TCR and pMHC. Panel **B**: The action of independent identically distributed random noises on the inputs of the TCR array can be conceived as the nominal threshold θ would be distributed on θ_1_, θ_2_, …, θ_N_ according to some noise distribution, symbolized by the red curve, with standard deviation σ_1_. The working function of the TCR array now can be summarized as to take random samples from the signal distribution with the aid of the threshold distribution. The quality of sampling and ultimately that of the information transmission from the signal distribution is dictated by the degree of overlap between the signal and threshold distributions. In Panel **B**, sampling is performed mainly from the middle of signal distribution corresponding to undersampling, the standard deviation of threshold (σ_1_) being much smaller than that of signal, σ_1_ < σ. Panel **C**: The sampling by noise is optimal, and the transmitted information is the maximal, when the standard deviation of noise (σ_2_) equals that of signal σ_2_ = σ. Here, the whole range of signal distribution is sampled, at the cost of reduced sampling from the right portion of the signal distribution. Panel **D**: Information transmission is also not optimal when the noise distribution is wider than that of signal, σ_3_ > σ. Here only a portion of the information transmitting capability or “channel capacity” of the TCR array as an information channel is utilized. Equivalently, due to the unnecessarily large width of threshold distribution, sampling density from the range of signal is reduced. From Panels **A**–**D** also the most important attribute of SSR can be read off, namely, the existence of special noise level, where the transmitted information is maximal, for a given array size.

Let us suppose that the signal to be detected, in our case, the affinity distribution in the pool of high affinity pMHCs, is Gaussian distributed around the mean θ with a standard deviation of σ. Let the threshold of each detector be the same nominal θ, i.e., equal to the signal mean (Figure 3 Panel A). Then the noise superposing on the signal at the input of the detectors can also be conceived as a modulation of the θ. As a result, the detector array behaves as a stochastic quantizer, taking samples from the signal distribution at N different threshold values, randomly changing in time according to a probability distribution determined by the nature of the noise. The accuracy of coding the input signal is dictated by the type of noise distribution as well as the number of detectors. Let us now suppose that the noise distribution is also Gaussian with the same mean as thesignal but with different standard deviations, as shown by the red curves in Figure 3 Panels B–D. Then sampling with narrow noise distribution, red curve with standard deviation σ_1_, predominantly happens at the middle of signal distribution (Figure 3 Panel B), resulting in a rather non-representative (or under-) sampling. Upon widening the noise distribution, sampling will gradually become more representative.The best-case scenario is when the noise standard deviation equals that of the signal (Figure 3 Panel C), the red curve with σ_2_. This is the point when the information transmission capability of the detector array is maximally utilized. When the noise standard deviation is higher than that of the signal, the red curve with σ_3_ (Figure 3 Panel D), although the whole range of the signal is sampled, sampling is with a smaller resolution, because detectors having too low or too high thresholds will not detect, due to the absence of the signal (over-sampling).

The degree of sensitivity and specificity of detection is reflected by the degree of similarity between the distributions of the output and input signals, described by the correlation (*ρ*) or mutual information (*I*) between them, according to the following formula enabling interconversion of the two [75]:

(1) $$I=-\log_{2}(1-\rho^{2})/2,$$

where *I* is measured in bits due to the logarithm of base 2, and *ρ* is between −1 and +1. Strictly speaking, Equation (1) is valid only for linear dependence of the output signal on the input signal when the correlation is a valid descriptor. For nonlinear dependencies information stays to be a valid descriptor, but correlation fails. Information can be taken as a kind of extension of correlation, remaining valid also for non-linear input-output signal dependencies.

As we saw, by increasing the noise level, this achieves a maximum. This is valid for all array sizes, or detector numbers (N > 2). As to the dependence on array size, the transmitted information (or correlation) increases with array size, independently of noise level [31]. As an output signal here, the temporal profile of the number of the phosphorylated ITAMs can be taken.

**Figure 4:**
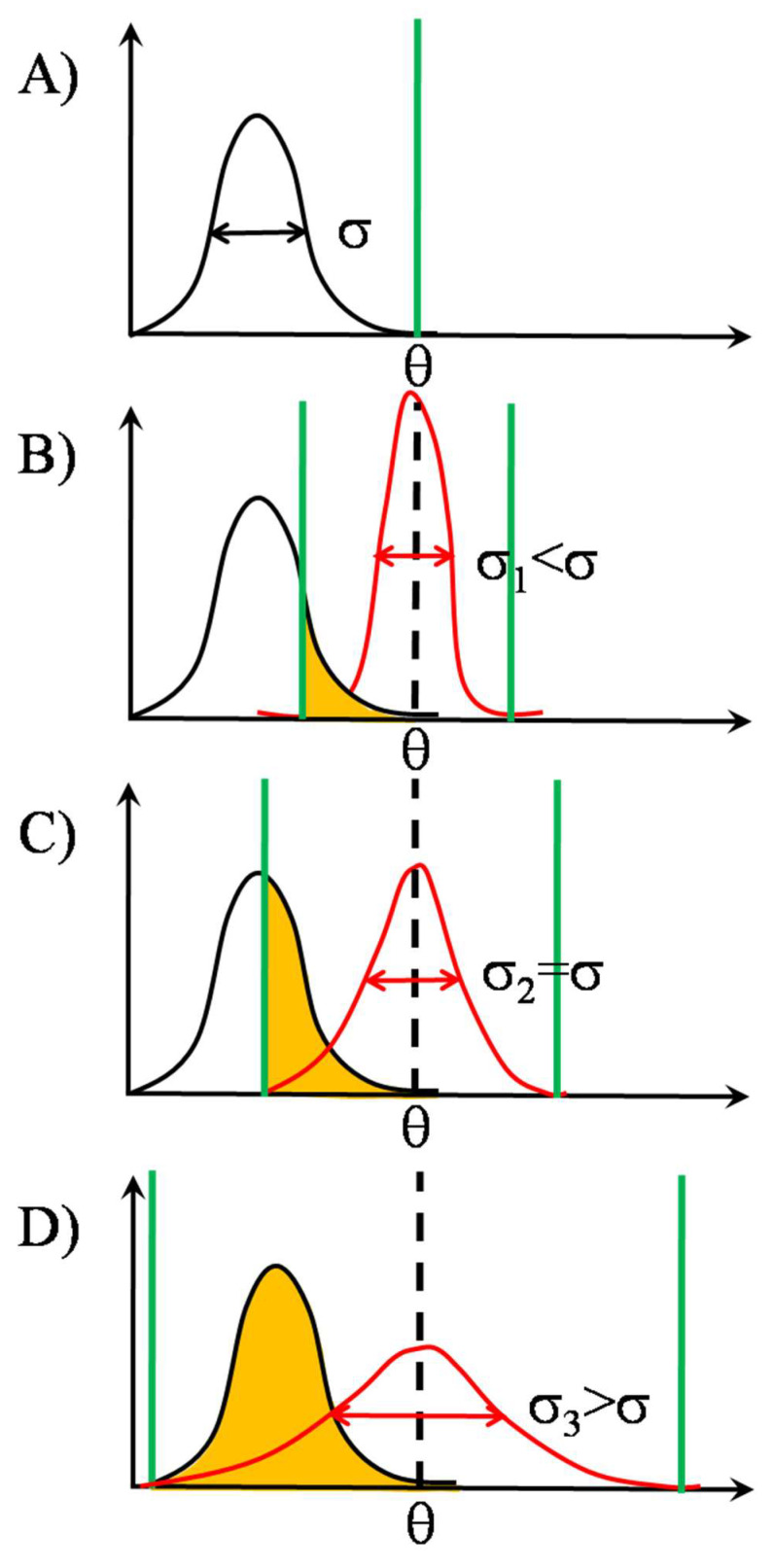
Types of gating with threshold distributions. Off-centered signal and threshold distributions, case I: signal mean smaller than threshold mean (sub-threshold gating). The effect of increasing noise level when the signal is entirely sub-threshold [31]. Information transmission is gradually improved by opening out the threshold distribution. At the end, although the whole signal range is sampled, the sampling density is reduced due to the shallowing of the threshold distribution. By judging from the shape of the overlap region, the symmetric signal distribution will be skewed to the left after the asymmetric sampling. Panel **A**: Nominal threshold level of individual detectors in the TCR array of Figure 2 is designated by θ. Its position is marked by a vertical green thick line on the horizontal axis of the detected signal, whose distribution is represented as a Gaussian black curve with standard deviation σ. The signal now is entirely subthreshold, because the whole distribution is located left to θ. Panel **B**: Upon the action of independent random noises at each input of the TCR array, the nominal threshold θ becomes distributed on θ_1_, θ_2_, … θ_N_ according to the noise distribution, symbolized by the red curve, with standard deviation σ_1_. Sampling is performed by the threshold distribution mainly from the right tail of signal distribution, marked by orange shading, corresponding to heavy undersampling, the standard deviation of threshold (σ_1_) being much smaller than that of signal, σ_3_ << σ. Panel **C**: By equalizing the widths of noise and signal distributions, σ_2_ = σ, sampling becomes better as judged from the larger orange overlap area (half the signal), but yet remaining undersampling. Panel **D**: Information transmission is the best, when the noise distribution becomes wider c.a. double of that of signal, σ_3_ = 2σ. Upon a further increase in the width of noise distribution, new empty thresholds left to the signal range tend to appear.

**Figure 5:**
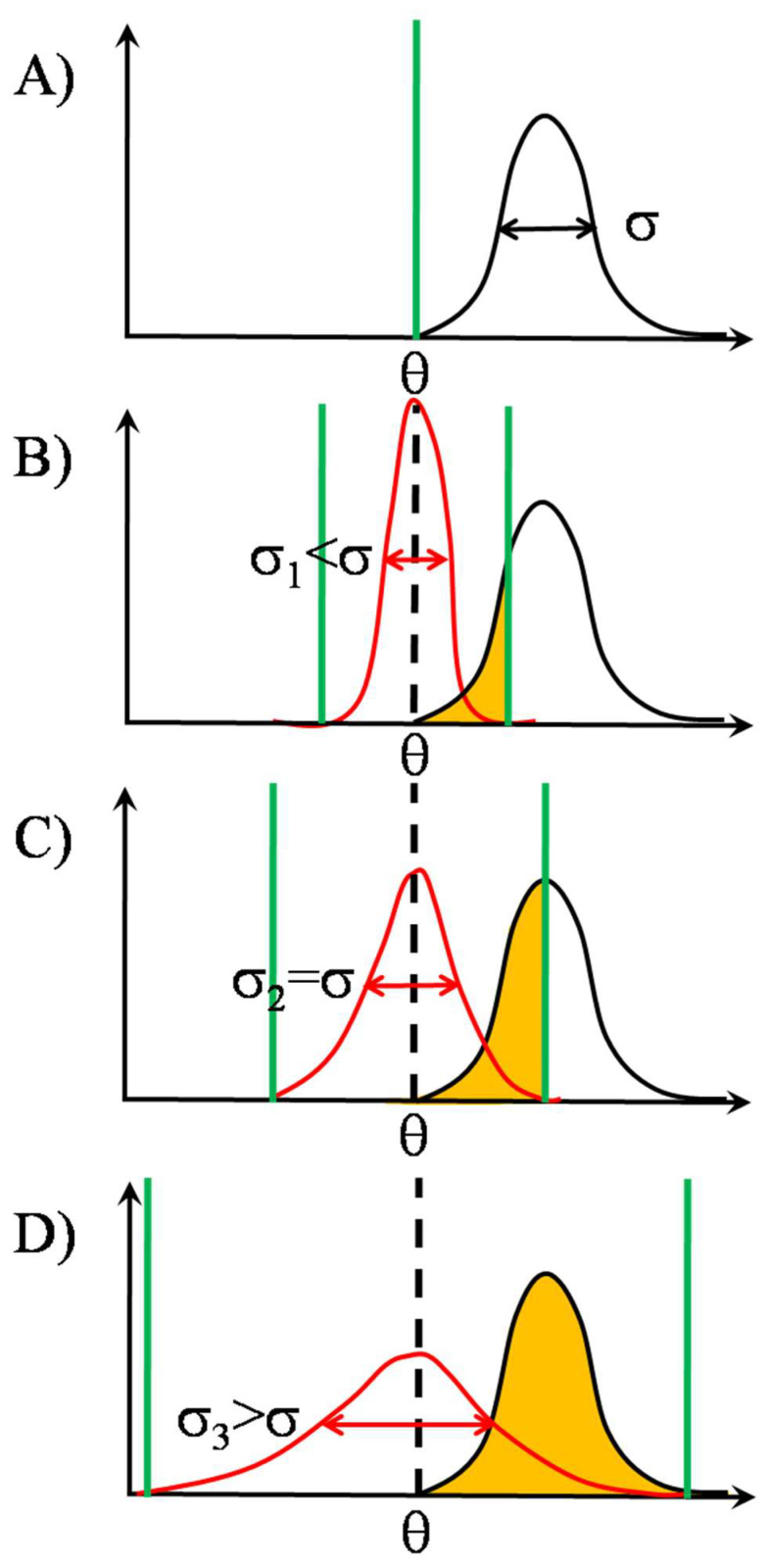
Types of gating with threshold distributions. Off-centered signal and threshold distributions, case II: signal mean larger than threshold mean (suprathreshold gating). The effect of increasing noise level when the signal is entirely suprathreshold [31]. Here only detrimental effects of noise on information transmission can be revealed. By gradually increasing the width of threshold distribution an increasing portion of the originally suprathreshold signal distribution becomes sub-threshold, resulting in a gradual undersampling of the left side of the signal distribution. Panel **A**: Nominal threshold level of individual detectors in the TCR array of Figure 2 is designated by θ. Its position is marked by a vertical green thick line on the horizontal axis of the detected signal, whose distribution is represented as a Gaussian black curve with standard deviation σ. The signal now is entirely suprathreshold, meaning maximal information transfer, because the whole distribution is right to θ. Panel **B**: Upon the action of independent random noises at each input of the TCR array, the nominal threshold θ becomes distributed on θ_1_, θ_2_, … θ_N_ according to the noise distribution, symbolized by the red curve, with standard deviation σ_1_. Sampling is preferentially performed now by the threshold distribution from the right tail of signal distribution, the complement of the orange shaded area, corresponding to undersampling, the standard deviation of threshold (σ_1_) being much smaller than that of signal, σ_1_ << σ. Panel **C**: By equalizing the widths of noise and signal distributions, σ_2_ = σ, sampling becomes worse, meaning heavier under-sampling, as judged from the larger orange overlap area (half the signal range). Panel **D**: Information transmission is the worst, when the width of the noise distribution becomes equal with the double of that of signal, $\sigma_{3}=2\cdot \sigma$.

Further examples of the SSR mechanism are illustrated in Figure 4 and Figure 5 for signals entirely sub- and supra-threshold in the absence of noise, respectively.

#### 2.2.3. Noise’s Nature

Because the ultimate requirement of signal transduction in TCR is an appropriate degree of conformational change, all factors resulting in the conformational heterogeneity of TCR can be designated as noise. Local variability of physico-chemical parameters of the lipid membrane, such as fluidity, cholesterol level, lipid constitution, and membrane potential, is a candidate. For example, it is known that certain ionic channels, such as voltage-gated ion channel K_v_1.3, participate in the formation of the immune synapse. Local activation of these channels might modulate the free energy requirement of the conformational change, leading to activation.

The phenomenon of SSR does not require that the individual TCR molecules be in close, or molecular proximity. The array model only assumes the presence of some receptors in the area of the immune synapse acting in synchrony. According to the information or correlation vs. receptor number curves, the increase in these quantities with receptor number is the steepest at low receptor numbers, with a gradual plateauing at high receptor numbers [31]. However, at the molecular level, contacting proximities of TCRs have also been observed, enabling local modulation of the availability of the ITAMs in the cytoplasmic tails of adjacent TCRs for phosphorylation.

### 2.3. Qualifying CARs with SR

Interaction between two cells is mediated by pattern recognition processes. As a special pattern recognition process, SR speaks about the presence of some degree of optimality. The system becomes the optimal state in the course of gradual ripening, via adaptations to the diversity of noise. Optimality, the presence of maxima in the information vs. noise level curves, also assumes the presence of negative feedback processes. With the natural TCR, signaling has the following two arms: The membrane proximal first arm, activated in a short time, is responsible for creating the local milieu, the boundary conditions, for SR to take place. This local milieu is related to the spatial proximities of TCRs towards each other and to other signaling molecules, ensuring amplification, threshold, and negative feedback. Then the second arm focuses the signaling to the center in an optimized manner, the degree of which is reflected by the presence of SR. Consequently, operation in ill or diseased conditions manifests itself in a reduced capability for SR. 

A quality index of engineered chimeric antigen receptors (CARs) might be the presence of SR [32,33,34,35,36,37]. SR develops through gradual adaptations to a vast number of diversities caused by the noise. Absence of SR might indicate a suboptimal functioning of CAR, either anemia for eliciting activating signal, when the first arm is present, but the second arm is missing, e.g., when threshold, negative feedback is too high. Or overshooting of the second arm, causing cytokine storm because of the malfunction of the first arm in ensuring adaptation, leading to low threshold and weak negative feedback. In our opinion, CAR systems should be engineered by monitoring the presence of SR at each stage of development.

The SSR is characterized by the existence of a maximum of the transmitted information vs. signal noise [31]. However, signal noise can also be interpreted as a diversity of threshold levels of the TCRs. The threshold level of the TCR is determined partly by the soft threshold built into the TCR structure and partly by the high tuning of this threshold by the coreceptor CD4/CD8 proximity to the TCR. The soft threshold actually means not a single sharp threshold, but rather a gradual transition from an inactive, capable of signaling along the membrane surface, to an active conformation of the TCR, capable of signaling towards the nucleus (Figure 1). Consequently, there is a threshold distribution naturally built into the TCR structure that can play the role of noise. This distribution is further widened by the above discussed physico-chemical parameters of the membrane.

Keeping in view of the above, when CAR systems are engineered, it might be advantageous to introduce a given degree of diversity either at the molecular level in a single cell or at the level of cell populations. At the molecular level, diversification in the different molecular regions, recognition, transmembrane, and intracellular, of the main signaling receptor can be imagined, equipped with a panel of artificial coreceptors. At the cellular level, this diversification can be achieved by transfection with a panel of CAR systems having different thresholds, resulting in mixed-CARs in a single cell. The beauty of SSR is that the same principles can be carried over from the level of the receptor array to the level of a cell population, with the individual cells as the detectors [41]. CAR mixing in this case might imply mixing of cells, each transfected with a differently thresholded CAR. Success might be anticipated based on the larger degree of adaptation, as described by the degree of developed SR.

## 3. Discussion

### 3.1. Two Levels of SR in Immune Recognition

Because TCR is a threshold detector (Figure 1), the occurrence of SR can be anticipated. As far as we know, the possible involvement of SR in immune recognition processes has first been raised by C. Wülfing et al. [43,44], noticing that noncognate pMHCs might help detection of cognate pMHCs by the TCR. SR can occur in immune recognition both at the single molecular and population levels.

(i) SR in single TCR

At the single TCR level, it is the conventional subthreshold SR with a role in facilitating detection of weak agonists (Figure 2 Panels A,B). An important circumstance is that the cognate pMHCs generally form extended homo-associations with other MHCs and hetero-associations with other, non-MHC receptors [1,2,3,4,5,10]. Simultaneous interactions of the TCR with MHC dimers or tetramers have also been described [10]. This implies that the scanning TCR should pick out the wanted cognate pMHC from a highly crowded field of noncognate pMHCs and another receptor. Molecular crowding has been shown to accelerate protein folding reactions, such as the conformational change of TCR, necessary for threshold crossing [76,77,78]. According to this folding picture, the momentarily or previous interactions of the TCR with the crowded neighborhood set the TCR into unspecific, subthreshold conformational oscillations at a lot of frequencies, from which a given, specifically loaded pMHC can select for, according to its vibrational eigenfrequency. The energy of the selected stored vibration at the eigenfrequency then adds to the energy of the vibration elicited by the given pMHC in the TCR. Otherwise, in the fluctuating, crowded environment, the scanning TCR takes up and stores energy in the form of conformational oscillations at many frequencies, amongst them the one necessary for a given cognate pMHC to induce a threshold-crossing conformational change. These stored oscillations can also be conceived as a natural factor contributing to the broadening of the threshold spectra of TCR, which is important in SSR.

### 3.2. SR in TCR and MHC Arrays: SSR

At the population level, it is SSR, with the role of optimal information transfer through the membrane (Figure 2 Panels C,D). Historically, the SSR phenomenon has been discovered in connection with efforts to improve cochlear implants’ operation [31,38]. Optimal information transfer here refers not only to ensuring an output signal proportional to the input, but also keeping the faithful temporal profile of the input. This capability of SSR can be quantitatively described by tools of information theory, such as mutual information, channel capacity, mean error distortion, and correlation [31]. These quantities can all be computed from the characteristics of signal and noise, or what is equivalent to that of the threshold. Optimality means that, there always, independently whether the signal is sub- or supra-threshold, exists a noise level where the above quantities assume their maxima or minima. In parallel, the functional relationship between the input and output, the “transfer function”, turns from non-linear, characteristic of a single TCR, to linear, characteristic of the TCR population. The degree of showing the SSR phenomenon essentially reflects the capability for adaptation by the system considered.

Further importance of SSR lies in that, via its capability of preserving temporal profile of input signal, it enables frequency encoding of input signal at the output. Besides size of the signal, its frequency content can also convey information to the nucleus, e.g., the expression of NF-κB is frequency encoded [79].

The MHC molecules on the presenting APCs are also clustered [1,2,3,4,5,10]. Although they are not of the type of bona-fide signaling receptors due to the short length of their cytoplasmic tails, nevertheless, some signaling capabilities have also been reported for them, e.g., in apoptosis [6,7,8,9]. Although the occurrence of SR can not be excluded in populations of MHCs on the presenting cell, first the thresholding properties of MHC should be clarified for this.

### 3.3. Exploiting SSR to Optimize Molecular Prostheses

Transgenic chimeric antigen receptors (CARs) are genetically engineered artificial receptors for restoring or improving functionality at the level of immune recognition in malfunctions such as cancer with a decreased level of pMHC [1,2,3,32,33,34,35,36,37]. They are custom-made synthetic receptors, genetically tailored from domains of TCR, coreceptors, and immunoglobulins, called monoclonal antibodies (mAbs), for catching antigenic peptides and eliciting T-cell activation. Sometimes they recognize pMHCs in the absence of coreceptors CD8 and CD4, implying that their recognition is not “MHC-restricted”. Until now, three generations of CARs have been developed. Applying them, major problems might be the missing tolerance (grafting), anemia for signaling or signal overshooting (cytokine storm) and mixing with wild-type receptors of the host, i.e., dilution [32,33,34,35,36,37].

Except for the mixing problem with wild type receptors, these issues can be dealt with in the framework of SR, similarly to the approach to cochlear implants [31,38]. Specificity and sensitivity can be balanced with careful planning of threshold and the extent of immune synapse, governed by factors such as CAR expression level on the cell surface. Since the quality of information transfer by SSR is largely governed by the extent of overlap between the signal and noise (or threshold) distributions, we expect that the threshold distribution of CAR systems is the main factor to be optimized for the signal characteristics—e.g., surface density, cluster morphology of pMHC, expression level of coreceptors—in the given malfunction. Adaptation and achieving adequate signaling without overshoot necessitates diversifying the thresholds of the individual CARs via, e.g., simultaneously applying a panel of CARs with gradually increasing thresholds. The goodness of a CAR system can be classified by the degree of the detectable SR, quantified with e.g., channel capacity, and coding efficiency [31]. The mere presence of SR, if any, reports on the degree of adaptation realized in the CAR system.

### 3.4. Förster (Fluorescence) Resonance Energy Transfer (FRET) as an Information Channel

Concerning the overlap of noise and signal distributions in SSR, an analogue overlap, quantified by the overlap integral J, between the donor emission and acceptor absorption spectra is the prerequisite for Förster-type resonance energy transfer (FRET) to take place [1,2,3,4,5,10]. The acceptor absorption spectrum corresponds to the noise distribution of SSR, which takes samples from the donor emission spectrum, which in turn corresponds to the signal distribution. The J overlap integral for FRET corresponds to the mutual information transferred by SSR [31]. This observation is plausible because both FRET and SSR are information transmission processes. However, FRET has the following other very important characteristics: its sharp distance dependence, on which its widespread biological application is based, in monitoring distances and morphology of receptor clusters [1,2,3,4,5,10]. A question arises now concerning the possible dependence of the SSR on the mutual proximities of the elements of the detector array, i.e., those of TCRs. These proximities probably might influence the noise distributions at the inputs of the detectors. The noise distributions might become correlated depending on the degree of coupling between the detectors, which might show some distance dependence, mediated by the membrane bilayer, in each other’s close (molecular) proximity.

### 3.5. SSR as an Analogue-to-Digital Signal Converter (ADC)

What is remarkable is that SSR acts as an analogue-to-digital converter, via sampling from a continuous analogue signal into a digital output (blue input signal in Figure 2 Panel D, and green-white-red histogram, respectively). The significance of digitalization is that digital signals have better error tolerance as compared to analogue (continuous) ones [31]. By utilizing this mechanism, information might reach the cell nucleus more safely.

### 3.6. Future Directions and Limitations of the SR Model

Based on the afore-discussed qualitative properties of the TCR-MHC system, building a detailed mathematical model might be approached. For this, the formalism given in [31] might be adequate. However, SSR is discussed in [31] in terms of an array of identical threshold detectors when each member of the array is influenced by the noise of the bathing environment independently. The condition of independence might be violated due to the close association of the detectors (receptors) when the detectors start to interact with each other.

### 3.7. Metastases Finding as a Problem of Weak “Fault Signal” Detection

A big challenge of cancer diagnosis is to reveal primary tumors or secondary ones (metastases) after surgical tumor removing when their size is the smallest. In this respect, approaches well elaborated in engineering practice for the detection of rare and weak fault signals (e.g., cracks) might be overtaken. These approaches are also based on SR [80,81]. Their essence is that the originally undetectable, primary weak fault signal is amplified by SR to a detectable level, reminiscent of the principle of polymerase chain reaction (PCR) in biotechnology.

## 4. Conclusions

Based on structural data, TCR has been shown to operate as a threshold detector. Noise-assisted signal detection and information transfer are described both at the single TCR level (SR) as well as at the TCR population level (SSR). Analogously to optimizing cochlear implants for better hearing, optimizing molecular prostheses such as CARs against cancer for better transmembrane signaling is suggested in the framework of SSR. A parallelism is noted between FRET and SSR concerning information transmission.

## Data Availability

Not applicable.

## Abbreviations

AFM	atomic force microscopy,
APC	antigen presenting cell, e.g., dendritic cell,
CAR	genetically engineered chimeric antigen receptor, composed of the variable and constant regions of the antigen recognising F_ab_ portion of antibody connected by a linker region, as well as a spacer and transmembrane region, and an intracytoplasmic domain containing 3–5 (signaling) sites, which can be phosphorylated, depending on the generation of CAR (1th–4th),
CD28	coreceptor (signal amplifier) on the side of TcR on a T cell in apposition to an APC, its counter receptor is CD80,
CD45/CD148/CD137	phophatases, dephosphorilate sites, which can be phophorylated, act against kinases,
CD8/CD4	coreceptors (signal amplifiers) CD8 and CD4 on T cells—killer and helper, respectively–, binding to MHCI and MHCII, respectively on an APC in apposition to a T cell,
cSMAC/pSMAC/dSMAC	central, peripheral, and distal supramolecular activation clusters, concentric zones—centripetally from inside to outside—constituting the immune synapse (IS) on T cells, rich in non-signaling TcRs
(cSMAC)	microclusters of signaling TcR and LFA-1 (pSMAC), and rich in CD45 (dSMAC),
FRET	Förster (or fluorescence) resonance energy transfer, occuring between an energy donor and an energy acceptor when their distance is between 1 nm and 10 nm,
ICAM-1/ICAM-2	intracellular adhesion molecules type 1 and type 2, on the surface of an APC, binding to LFA-1/LFA-3 on the recognising T cell,
IS	immune synapse, consisting of concentric zones called cSMAC, pSMAC, and dSMAC,
ITAM	tyrosine based activation motif, which can be phosphorylated, or docking site for tyrosine kinases Lck, Src,
Lck	Src tyrosine kinase p65lck,
LFA-1/LFA-3	leukocyte function associated antigen type 1 and type 3 on the side of TCR on the recognising T cell, its counter receptor on the APC is ICAM-1/ICAM-2, capable also for signaling,
MHCI/MHCII	Major Hystocompatibility Gene Complex products I and II, presenting antigen of the surface of an APC towards effector cells, killer and helper T cells, respectively,
pMHC	MHC molecule presenting short—8–9 amino acids long—peptide fragment of antigen on the surface of APC towards the recognising receptor TCR on the surface of an effector (killer, helper, memory) T-cell,
SR	stochastic resonance,
SSR	supra-threshold stochastic resonance,
TcR	T-cell receptor—composed of a heterodimer of TCRα and TCRβ (briefly αβ) recognising the antigen, a long signaling domain TCRζζ (briefly ζζ) containing 6 phosphorilatable sites (ITAMs) and CD3γε (briefly γε), CD3δε (briefly δε) signaling domains each containing 2 sites, which can be phosphorilated—binding to peptide-loaded MHCs on the surface of an APC,
TEM	transmission electron microscopy,
TM4SF	transmembrane-4 family protein, accessorical, adaptor protein spanning the membrane four times.
